# Identification of AP002498.1 and LINC01871 as prognostic biomarkers and therapeutic targets for distant metastasis of colorectal adenocarcinoma

**DOI:** 10.1002/cam4.6823

**Published:** 2023-12-11

**Authors:** Na Wu, Jingyi Chen, Tingru Lin, Zhaohui Zhong, Mei Li, Yimeng Yu, Jingzhu Guo, Weidong Yu

**Affiliations:** ^1^ Department of Central Laboratory and Institute of Clinical Molecular Biology Peking University People's Hospital Beijing China; ^2^ Department of Gastroenterology Peking University People's Hospital Beijing China; ^3^ Department of General Surgery Peking University People's Hospital Beijing China; ^4^ Department of Pediatric Peking University People's Hospital Beijing China

**Keywords:** AP002498.1/miR‐4443/SERPINA1 axis, colorectal adenocarcinoma, LINC01871/miR‐4644 and miR‐185‐5p/GNLY axes, liver metastases, prognostic biomarker, therapeutic target

## Abstract

**Background:**

Increasing evidence suggests that lncRNA (Long non‐coding RNA, lncRNA)‐mediated ceRNA (competing endogenous RNA, ceRNA) networks are involved in the occurrence and progression of colorectal cancer (CRC). However, the roles of the lncRNA–miRNA–mRNA ceRNA network in distant metastasis of CRC are still unclear.

**Methods:**

In this study, we constructed a specific ceRNA network to identify potential biomarkers and therapeutic targets for distant metastasis of CRC. Specifically, RNA‐Seq data from The Cancer Genome Atlas (TCGA) were used to screen for differentially expressed lncRNAs (DElncRNAs) and mRNAs (DEmRNAs) related to metastasis. After validation and selection by qRT–PCR and univariate and multivariate analysis of the metastasis‐ and prognosis‐related lncRNAs, the regulated microRNAs (miRNAs) and coexpressed mRNAs were used to construct a ceRNA network for distant metastasis of CRC.

**Results:**

Two key distant metastasis‐related DElncRNAs, AP002498.1 and LINC01871, were identified by univariate and multivariate analysis in combination with analyses of clinical data and expression levels. Furthermore, lncRNA‐associated ceRNA subnetworks were constructed from the predicted miRNAs and 13 coexpressed DEmRNAs (SERPINA1, ITLN1, REG4, L1TD1, IGFALS, MUC5B, CIITA, CXCL9, CXCL10, GBP4, GNLY, IDO1, and NOS2). The AP002498.1‐ and LINC01871‐associated ceRNA subnetworks regulated the expression of the target genes SERPINA1 and MUC5B and GNLY, respectively, through the associated miRNAs.

**Conclusion:**

The DElncRNA AP002498.1 and the LINC01871/miR‐4644 and miR‐185‐5p/GNLY axes were identified as being closely associated with distant metastasis and could represent independent prognostic biomarkers or therapeutic targets in colorectal adenocarcinoma.

## 1 INTRODUCTION

Colorectal cancer (CRC) is one of the most common malignant tumors worldwide and has the third highest annual new incidence and mortality among all malignant tumors.^1^ CRC is the second most common malignancy in China. The incidence rate of CRC has increased rapidly in recent years and is currently ranked second among all malignancies, with the fifth highest mortality.^2^ Metastasis is the leading cause of CRC‐related death.^1^, ^2^

Approximately 20% of CRC patients have distant metastases at diagnosis,^3^ frequently in the liver, and most CRC patients eventually develop distant metastases.^4^ Distant metastases develop when CRC cancer cells leave the primary site, intravasate into the blood and lymphatic circulation by degrading the extracellular matrix, and then invade specific organs where they are seed and grow progressively.^5^, ^6^, ^7^, ^8^, ^9^ Although chemotherapy and targeted drugs have greatly improved the efficacy of CRC therapy, 35% of CRC patients still develop liver metastases.^3^ Therefore, it is imperative to understand the molecular mechanism(s) underlying the development of distant metastasis in patients with CRC and identify novel biomarkers and therapeutic targets to improve the prognosis of CRC patients.^4^, ^5^, ^7^, ^9^

Long noncoding RNAs (lncRNAs) (containing >200 nucleotides)^10^, ^11^ are involved in the initiation and progression of malignant tumors, including CRC.^12^, ^13^, ^14^ Studies have highlighted that lncRNAs can bind to sites in microRNAs as competing endogenous RNAs (ceRNAs), thereby regulating the expression of mRNAs and their target genes. These lncRNA‐associated ceRNAs have been suggested to play an important role in cancer initiation and progression.^15^ However, the regulatory mechanisms and prognostic roles of lncRNA‐mediated ceRNA networks in distant metastasis of CRC have not been elucidated.

**FIGURE 1:**
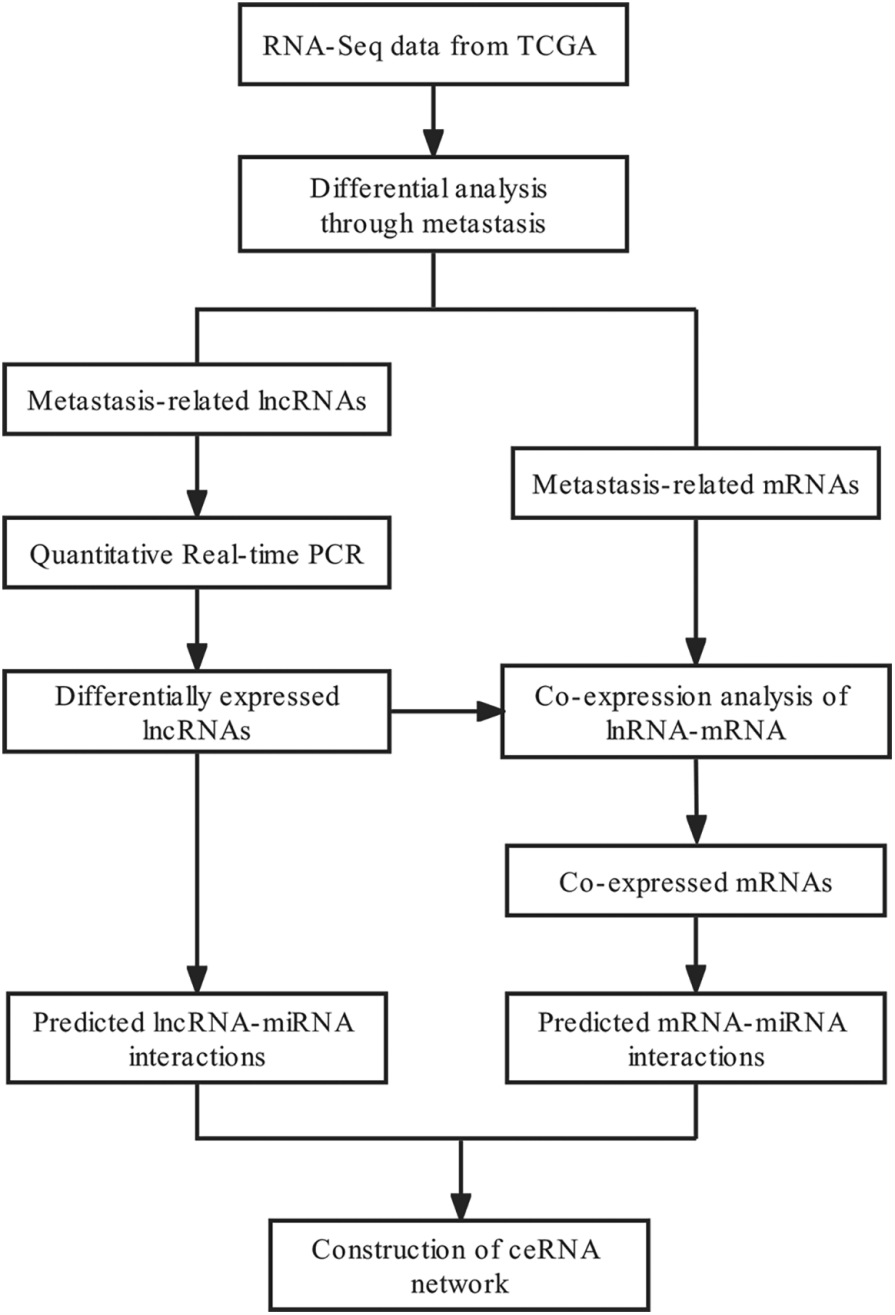
Flowchart for the identification and analysis of distant metastasis‐related and prognostic lncRNAs in the ceRNA network.

In the present study, we identified two key differentially expressed lncRNAs (DElncRNAs) and the associated regulated mRNAs (DEmRNAs) related to CRC metastasis using The Cancer Genome Atlas (TCGA) database. The CRC metastasis‐ and prognosis‐related lncRNAs and the coexpressed mRNAs were validated by quantitative reverse transcription–polymerase chain reaction (qRT–PCR). Univariate and multivariate analyses were performed in combination with analyses of clinical data and the expression levels of lncRNAs and the associated mRNAs, and the results were incorporated for construction of the ceRNA network. Finally, the axes involving the DElncRNAs AP002498.1 and LINC01871/miR‐4644 or miR‐185‐5p/GNLY were identified to be closely associated with distant metastasis and could be promising independent prognostic biomarkers or therapeutic targets for distant metastasis of CRC (Figure 1).

## 2 MATERIALS AND METHODS

### 2.1 Patient characteristics and sample selection

Colon tumor tissues from 46 CRC patients were collected at Peking University People's Hospital. This research project was approved by the Research Ethics Committee of Peking University People's Hospital (Approval No: 2019PHB028‐01). All of the subjects signed informed consent forms prior to participation. The clinicopathological details of the patients are listed in Table S1. The tumor‐node‐metastasis (TNM) stage was determined according to the Union for International Cancer Control (UICC) staging system (2016 version). The patients included were 25 men (54.3%) and 21 women (45.7%), with an average age of 71 years (range 31–99 years), who had a confirmed diagnosis of colon carcinoma based on the histopathology of the resected material. Twenty patients had lymph node metastases, and six had distant metastases. The lncRNA expression levels in the tumor tissues were measured by RT–PCR.

### 2.2 RNA isolation and complementary DNA (cDNA) preparation

A single core tumor biopsy sample with a diameter greater than 5 cm was collected from each CRC patient. Total RNA was isolated using the QIAGEN miRNeasy Mini Kit (QIAGEN, Germany) according to the manufacturer's instructions. Total RNA was reverse‐transcribed into cDNA using the PrimeScriptTM RT Reagent Kit (Takara, Japan). Each reaction mixture (10 μL) contained 500 ng of RNA, 5× master mix, and reverse transcriptase.

### 2.3 Quantitative reverse transcription PCR (qRT–PCR) analysis of lncRNAs

qRT‐PCR was performed in triplicate according to the manufacturer's instructions. Briefly, each reaction mixture (20 μL) contained 100 ng of cDNA and SYBR Green Real‐time PCR Master Mix (Toyobo, Japan). Relative quantification of lncRNA levels was performed using GAPDH as the control gene (forward primer, 5′‐AATGAAGGGGTCATTGATGG‐3′; reverse primer, 5′‐AAGGTGAAGGTCGGAGTCAA‐3′).^16^ DElncRNA primer‐related information is listed in Table S3.

### 2.4 Data source and preparation of TCGA data

The raw RNA‐seq data and clinical information associated with 548 CRC samples were downloaded from the TCGA database (https://cancergenome.nih.gov/). The count reads file and FPKM file downloaded from TCGA. Then, we used the “edgeR” package in R software to standardize the data and screen the differentially expressed RNAs. The Ensemble IDs in the standardized data were mapped to symbol IDs, which were used in subsequent data analysis. The clinicopathological details for the represented patients are presented in Table S2. The GENCODE database (https://www.gencodegenes.org/)^17^ was used to annotate the lncRNAs and protein‐coding RNAs. The data were extracted from the TCGA database according to the publication guidelines strictly approved by TCGA.

### 2.5 Identification and correlation analysis of metastasis‐related DElncRNAs and DEmRNAs

The lncRNA and mRNA expression profiles were divided into the metastasis and nonmetastasis groups based on the distant metastasis status (M stage) of the associated patient. The R package “edgeR” was used to identify the DElncRNAs and DEmRNAs between the metastasis and nonmetastasis groups.^18^ The inclusion criteria were |log2‐fold‐change| > 1 and adjusted *p* < 0.05. Six metastasis‐related DElncRNAs were identified, and the hub metastasis‐related DElncRNAs were selected based on the qRT–PCR results and the chi‐squared test, which was used to determine potential correlations between the clinicopathological characteristics and the lncRNA expression levels. The correlation coefficients between the metastasis‐related lncRNAs and mRNAs were calculated based on the lncRNA and mRNA expression levels using Pearson correlation analysis. The lncRNA–mRNA pairs with Pearson correlation coefficients >0.4 were considered to have coexpression relationships.

### 2.6 Prediction of lncRNA–miRNA and mRNA–miRNA pairs

We screened metastasis‐related mRNAs for positive correlations with the lncRNAs. Survival and univariate analyses (chi‐squared test) were further performed to validate the key mRNAs. These validated mRNAs were analyzed with TargetScan (http://www.targetscan.org/) to predict possible binding to miRNAs. In parallel, the miRDB (http://www.mirdb.org/)^19^ database was used to predict lncRNA–miRNA pairs. The prominent miRNAs that could regulate both lncRNAs and mRNAs, as identified by correlation analysis, were selected to construct the ceRNA network.

### 2.7 Biotinylated microRNA pull‐down assay

The miRNA of interest labeled with biotin at the 3′ end and a scrambled control miRNA were commercially synthesized. Cells were seeded in a 10 cm tissue culture dish 1 day before transfection. Forty‐eight hours post‐transfection with the control miRNA and 3′ biotin‐labeled miRNA, cell lysates were harvested, and pull‐down, RNA isolation, and RT–PCR were then performed according to a previous study.^20^, ^21^

### 2.8 Statistical analysis

SPSS version 22.0 (SPSS, Chicago, IL, USA) and GraphPad Prism 8.0 (La Jolla, CA, USA) software were used for statistical analysis. Data from all quantitative assays are presented as the means ± standard deviations (SDs). Comparisons between study groups were performed using two‐tailed Student's *t*‐test or the chi‐squared test. The Kaplan–Meier plots display the proportions of surviving patients (overall survival [OS] and disease‐free survival [DFS]) with respect to the length of follow‐up in months. To further perform survival analysis and the comparisons between clinicopathological parameters and the lncRNA/mRNA expression, 325 samples with full and sound clinical information, including age, sex, TNM stage, OS months, OS status, and DFS months, DFS status were included, shown in Table S6. Values lower than the median were considered to indicate low expression, and those greater than the median were considered to indicate high expression. *p* < 0.05 was considered to indicate a statistically significant difference.

## 3 RESULTS

### 3.1 DElncRNAs between patients with and without liver metastasis

**FIGURE 2:**
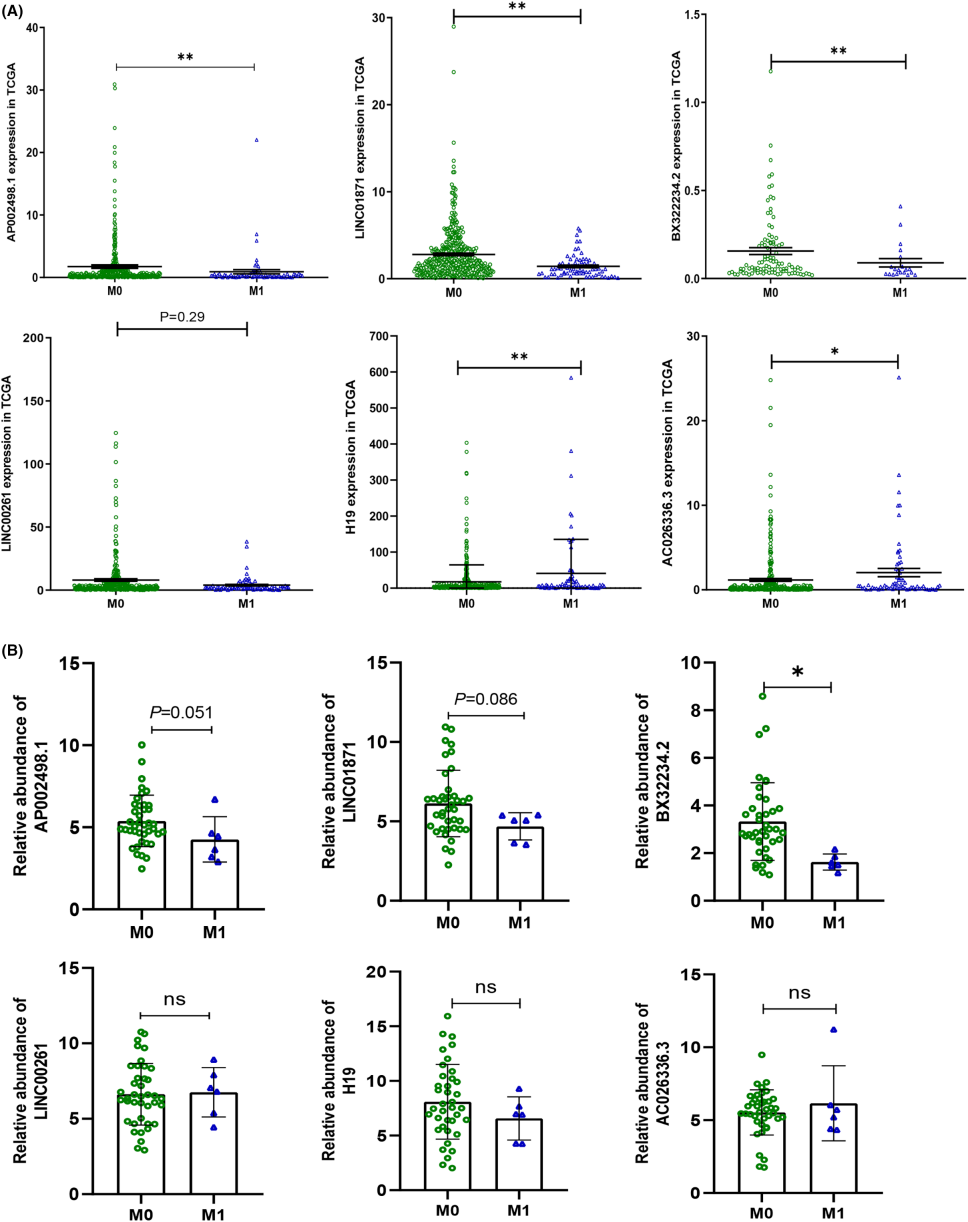
Distant metastasis feature analysis and validation of the six DElncRNAs. (A) Correlations of lncRNA expression with pathological M stage. (B) qRT–PCR validation of the six DElncRNAs in 46 clinical CRC tissue samples. *p < 0.05, **p < 0.01.

The expression profiles of 14,166 lncRNAs were obtained from the transcriptome profiling data of CRC patients in the TCGA dataset. Differentially expressed RNAs were identified between patients with and without distant metastasis. The clinicopathological characteristics of the 548 patients represented in the TCGA dataset are shown in Table S2. We identified six DElncRNAs based on the M stage (Figure 2A). AP002498.1, LINC01871, BX322234.2, and LINC00261 were downregulated in patients with distant metastasis, while H19 and AC026336.3 were upregulated.

### 3.2 Evaluation and verification of lncRNA expression and clinicopathological characteristics

To verify the findings from the TCGA dataset analysis, the expression levels of six DElncRNAs were evaluated in 46 colon tumor tissues from Chinese CRC patients using qRT–PCR (Figure 2B). The detailed clinical characteristics of the 46 CRC patients recruited from Peking University People's Hospital are shown in Table S1. The expression levels of the lncRNAs AP002498.1 and BX322234.2 were significantly decreased in patients with distant metastasis compared to those without distant metastasis. There was also a trend toward a decreased LINC001871 level (*p* = 0.086).

The correlations between the six DElncRNAs and various clinicopathological parameters (e.g., age, sex, TNM stage, tumor stage, lymph node metastasis status, and distant metastasis status) were explored using the chi‐squared test (Table 1). The lncRNA AP002498.1 level was significantly correlated with lymph node metastasis (*p* < 0.05) and positively related to distant metastasis (*p* = 0.189). The LINC01871 and BX322234.2 levels were significantly correlated with distant metastasis (*p* = 0.029 and *p* = 0.029). Low levels of AP002498.1 and LNC01871 were significantly associated with high probabilities of lymph node and distant metastasis, indicating a poor outcome for this group of patients in the CRC cohort. The levels of H19, LINC00261, and AC026336.3 did not correlate with any of the clinicopathological characteristics.

**TABLE 1:** Correlations between clinicopathological parameters and the expression of six DElncRNAs in 46 colon cancer patients.

Clinicopatho‐logical parameter	No.	AP002498.1 level	χ^2^	*p* value	LINC01871 level	χ^2^	*p* value	BX322234.2 level	χ^2^	*p* value	H19 level	χ^2^	*p* value	LINC00261 level	χ^2^	*p* value	AC026336.3 level	χ^2^	*p* value
		Low/high			Low/high			Low/high			Low/high			Low/high			Low/High		
Total	46	23/23			23/23			23/23			23/23			23/23			23/23		
Age																			
<65	13	7/6	0.11	0.74	8/5	0.97	0.33	7/6	0.11	0.74	6/7	0.11	0.74	8/5	0.97	0.33	5/8	0.97	0.33
≥65	33	16/17			15/18			16/17			17/16			15/18			18/15		
Sex																			
Male	25	10/15	2.19	0.14	12/13	0.088	0.77	10/15	2.19	0.14	11/14	0.79	0.38	14/11	0.79	0.38	12/13	0.09	0.77
Female	21	13/8			11/10			13/8			12/9			9/12			11/10		
TNM stage																			
0–II	24	8/16	1.04	0.31	10/14	1.39	0.24	11/13	0.35	0.56	12/12	0	1	11/13	0.35	0.56	13/11	0.35	0.56
III–IV	22	15/7			13/9			12/10			11/11			12/10			10/12		
Tumor stage																			
0–II	9	4/5	0.11	0.74	3/6	1.24	0.46	4/5	0	1	6/3	1.24	0.27	3/6	1.24	0.46	3/6	1.24	0.46
III–IV	37	19/18			20/17			19/18			17/20			20/17			20/17		
Lymph node metastasis status																			
Negative	26	9/17	5.67	**0.017** *	13/13	0	1	13/13	0	1	13/13	0	1	11/15	1.42	0.23	16/10	3.18	0.07
Positive	20	14/6			10/10			10/10			10/10			12/8			7/13		
Distant Metastasis status																			
Negative	40	18/22	3.07	0.19	17/23	6.9	**0.029** *	17/23	6.9	**0.029** *	19/21	0.77	0.66	21/19	0.77	0.38	20/20	0	1
Positive	6	5/1			6/0			6/0			4/2			2/4			3/3		

* *p* < 0.05,

** *p* < 0.01.

### 3.3 Analysis of independent prognostic factors and correlations with clinical characteristics in the TCGA dataset

Univariate analysis was performed to further validate the correlations of the two prognostic risk‐related lncRNAs (AP002498.1 and LINC01871) and clinicopathological characteristics in the TCGA dataset (Tables 2 and 3). LNC01871 had a significant protective effect on TNM stage, tumor stage, and distant metastasis in the TCGA CRC dataset (Table 3).

**TABLE 2:** Correlations between clinicopathological parameters and the three DEmRNAs coexpressed with AP002498.1 in the TCGA dataset.

Clinicopatho‐logical parameter	No.	AP002498.1 level	χ2	*p* value	SERPINA1 level	χ2	*p* value	ITLN1 level	χ2	*p* value	REG4 level	χ2	*p* value
		Low/high			Low/high			Low/high			Low/high		
Total	325	163/162			163/162			163/162			163/162		
Age	325												
<65	159	76/83	0.69	0.41	78/81	0.15	0.7	80/79	0.003	0.96	81/78	0.078	0.78
≥65	166	87/79			85/81			83/83			82/84		
Sex	325												
Male	178	90/88	0.026	0.87	91/87	0.15	0.7	96/82	2.25	0.13	92/86	0.37	0.54
Female	147	73/74			72/75			67/80			71/76		
TNM stage	311^a^												
0–II	173	80/93	2.02	0.16	76/97	5.45	**0.02** *	82/91	1.48	0.22	74/99	7.09	**0.008** **
III–IV	138	75/63			79/59			75/63			80/58		
Tumor stage	325												
0–II	57	28/29	0.029	0.86	21/36	4.9	**0.027** *	25/22	0.046	0.83	27/30	0.22	0.64
III–IV	268	135/133			142/126			138/130			136/132		
Lymph node metastasis status	324^a^												
Negative	185	87/98	1.53	0.22	84/101	3.64	0.056	88/97	1.02	0.31	81/104	6.67	**0.01** *
Positive	139	75/64			78/61			74/65			81/58		
Distant Metastasis status	265^a^												
Negative	222	110/112	2.53	0.11	105/117	7.28	**0.007** **	111/111	3.3	0.069	104/118	4.81	**0.028** *
Positive	43	27/16			30/13			28/15			28/15		

^a^ Clinical data missing, unreliable or lost to follow‐up.

* *p* < 0.05,

** *p* < 0.01.

**TABLE 3:** Correlations between clinicopathological parameters and the three DEmRNAs coexpressed with LINC01871 in the TCGA dataset.

Clinicopatho‐logical parameter	No.	LINC01871 level	χ2	*p* value	IDO1 level	χ2	*p* value	CXCL10 level	χ2	*p* value	GBP4 level	χ2	*p* value
		Low/high			Low/High			Low/high			Low/high		
Total	325	163/162			163/162			163/162			163/162		
Age	325												
<65	159	79/80	0.027	0.87	83/76	0.078	0.781	88/71	3.357	0.067	81/78	0.078	0.78
≥65	166	84/82			80/86			75/91			82/84		
Sex	325												
Male	178	90/88	0.026	0.87	87/91	0.148	0.7	89/89	0.004	0.951	91/87	0.15	0.7
Female	147	73/74			76/71			74/73			72/75		
TNM stage	311^a^												
0–II	173	76/97	8.08	**0.004** **	80/93	4.982	**0.026** *	77/96	4.982	**0.026** *	77/96	4.98	**0.026** *
III–IV	138	83/55			76/62			79/59			79/59		
Tumor stage	325												
0–II	57	22/35	3.69	0.055	28/29	0.215	0.643	30/27	0.17	0.68	27/30	0.22	0.64
III–IV	268	141/127			135/133			133/135			136/132		
Lymph node metastasis status	324^a^												
Negative	185	82/103	6.18	**0.013** *	87/98	4.147	**0.042** *	83/102	4.548	**0.043** *	84/101	4.15	**0.042** *
Positive	139	81/58			76/63			79/60			79/60		
Distant Metastasis status	265^a^												
Negative	222	110/112	63.38	**0.000** **	104/118	7.87	**0.005** **	104/118	3.663	0.056	103/119	7.87	**0.005** **
Positive	43	30/13			29/14			27/16			30/13		

^a^ Clinical data missing, unreliable or lost to follow‐up.

* *p* < 0.05,

** *p* < 0.01.

**FIGURE 3:**
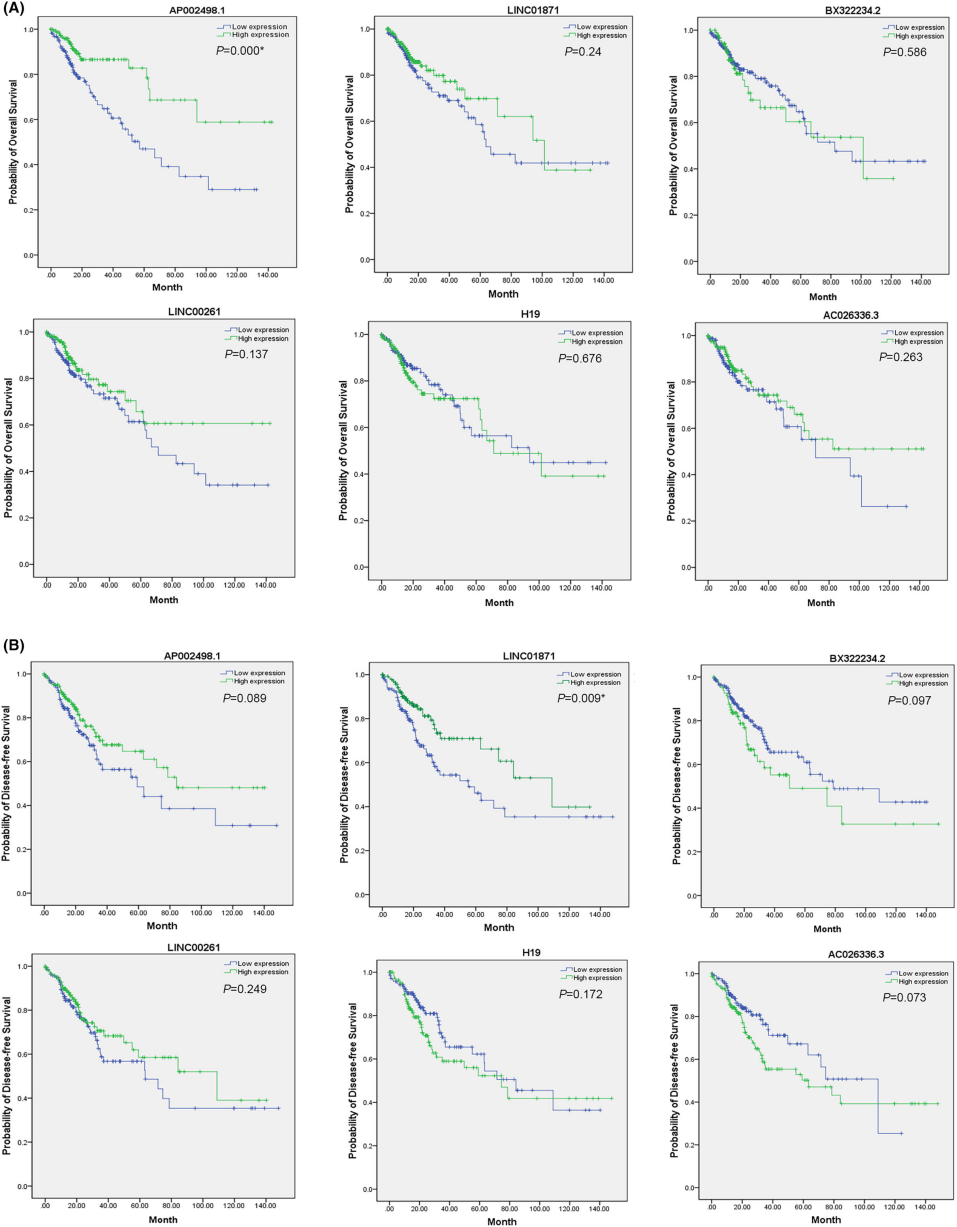
Kaplan–Meier survival curves based on the expression of the 6 DElncRNAs related to overall survival (OS) and disease‐free survival (DFS). (A) (OS) AP002498.1 was negatively correlated with OS, while LINC01871, BX322234,2, LINC00261, H19, and AC026336.3 were not correlated with OS. (B) (DFS) LINC01871 was negatively correlated with DFS, while AP002498.1, BX322234.2, LINC00261, H19, and AC026336.3 were not correlated with DFS. The green lines represent the high‐expression groups, and the blue lines represent the low‐expression groups. *p < 0.05, **p < 0.01.

Kaplan–Meier survival analysis was also performed based on the six DElncRNAs. The result of the OS and disease‐free survival analyses based on the six DElncRNAs are presented in Figure 3A,B, respectively. Among the six DElncRNAs, AP002498.1 was significantly linked with the prognosis of CRC patients (*p* = 0.000). Specifically, patients with low AP002498.1 levels had shorter OS times, suggesting a potential protective effect of AP002498.1 against distant metastasis. In addition, reduced LINC01871 levels were associated with a decreased disease‐free survival time (*p* = 0.009). Considering these results collectively with the RT–PCR results, these two DElncRNAs were identified as key lncRNAs in patients with distant metastasis.

Multivariate analysis was performed using a Cox regression model with the inclusion of all factors impacting patient prognosis (e.g., key lncRNA levels, age, sex, TNM stage, tumor stage, lymph node metastasis status, and distant metastasis status). In addition to age and TNM stage, AP002498.1 was an independent indicator of prognosis (*p* = 0.016; odds ratio = −0.823; 95% CI = 0.224–0.860).

### 3.4 Coexpression relationships between metastasis‐related lncRNAs and mRNAs

**FIGURE 4:**
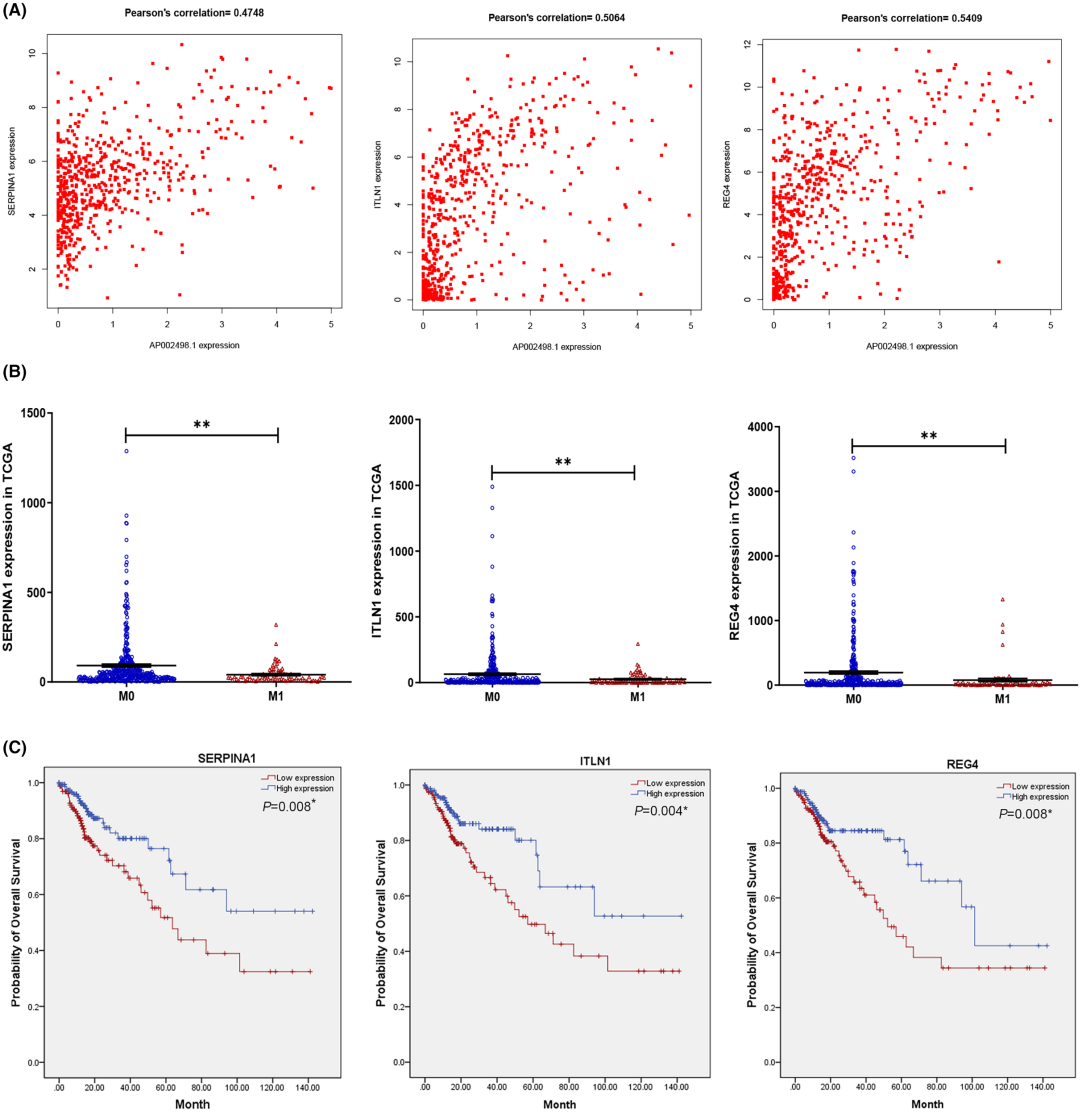
AP002498.1 and its paired coexpressed mRNAs. (A) Pearson correlation analysis between AP002498.1 and its coexpressed mRNAs (SERPINA1, ITLN1, and REG4). (B) Correlations of SERPINA1, ITLN1, and REG4 expression with pathologic M stage. (C) Kaplan–Meier survival curves based on the expression of the SERPINA1, ITLN1, and REG43 genes related to overall survival (OS). *p < 0.05, **p < 0.01.

The expression profiles of 19,645 mRNAs were acquired from the TCGA database, and 47 DEmRNAs (11 upregulated and 36 downregulated) were identified based on the distant metastasis stage (Figure S1). Pearson correlation analysis was then performed to evaluate the coexpression relationships between the metastasis‐related mRNAs and the two key DElncRNAs. The expression of AP002498.1 was positively correlated with that of six DEmRNAs (SERPINA1, ITLN1, REG4, L1TD1, IGFALS, and MUC5B) (Figures 4A and S2A), which were downregulated in patients with liver metastases (Figures 4B and S2B). Based on the survival analysis, SERPINA1, ITLN1, and REG4 might be significant protective prognostic factors for CRC, because high expression levels of these genes were significantly associated with longer OS times (Figure 4C). Moreover, we analyzed the associations between mRNA expression levels and the clinicopathological variables used to describe CRC progression in the 325 CRC patients with full and reliable clinical data from the cohort of 548 patients represented in the TCGA dataset. By the chi square test, SERPINA1 expression was found to be highly negatively correlated with TNM stage, tumor stage, and distant metastasis (*p* = 0.020, 0.027, and 0.007, respectively), indicating that lower SERPINA1 mRNA expression levels were associated with poorer outcomes for CRC patients in the TCGA dataset (Table 2). Of interest, ITLN1, REG4 and MUC5B were also identified as protective factors by univariate analysis. However, L1TD1 and IGFAL had no significant independent prognostic effects, based on this same analysis (Table S4). Based on the survival analysis and chi square test results, SERPINA1, ITLN1, REG4, and MUC5B were identified as hub AP002498.1‐related mRNAs.

**FIGURE 5:**
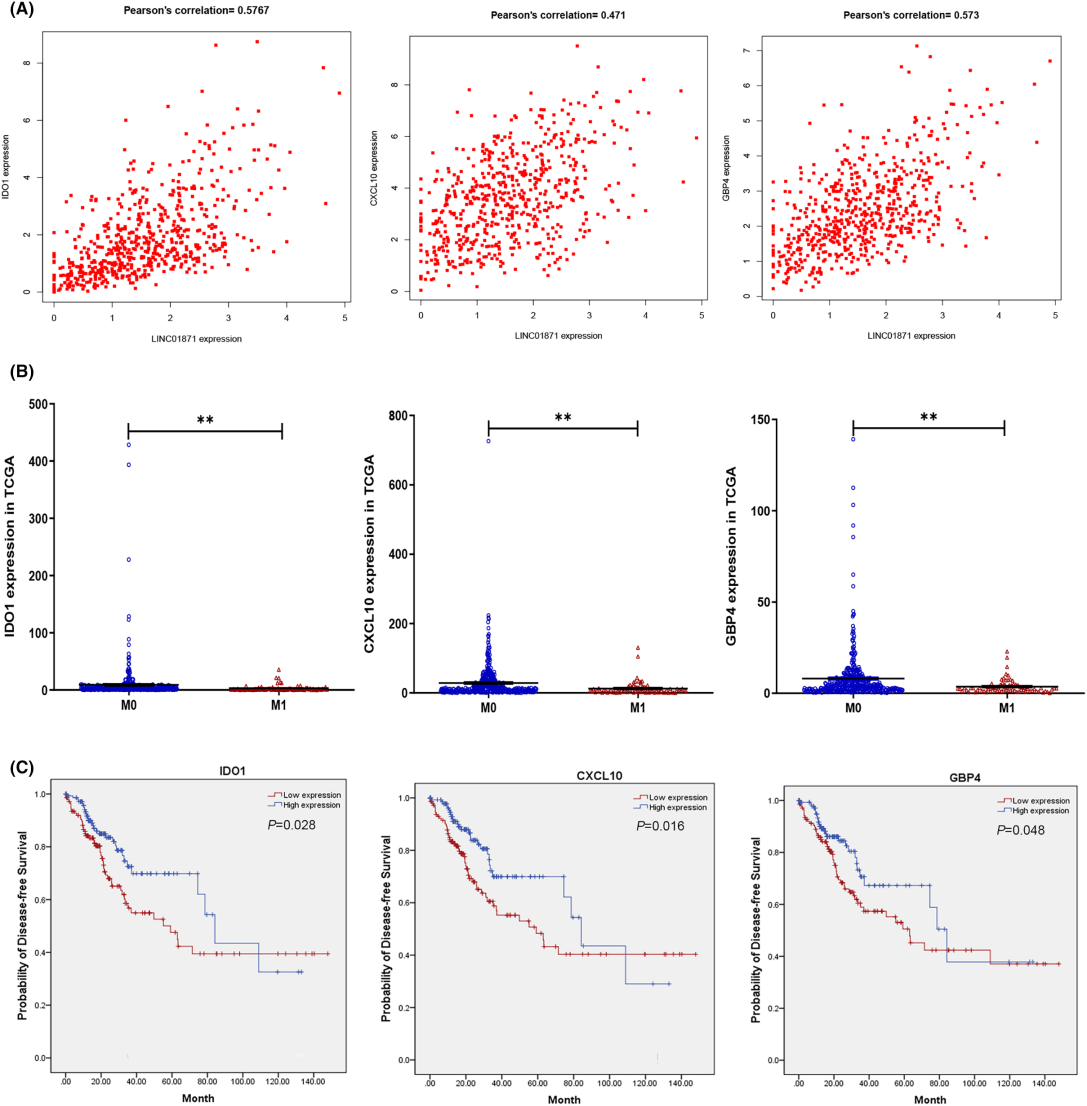
LINC01871 and its paired coexpressed mRNAs. (A) Pearson correlation analysis between LINC01871 and its coexpressed mRNAs (IDO1, CXCL10, and GBP4). (B) Correlations of IDO1, CXCL10, and GBP4 expression with pathologic M stage. (C) Kaplan–Meier survival curves based on the expression of the IDO1, CXCL10, and GBP4 genes related to disease‐free survival (DFS). *p < 0.05, **p < 0.01.

No DEmRNAs were positively correlated with BX322234.2, whereas LINC01871 was positively correlated with seven mRNAs (IDO1, CXCL10, GBP4, CIITA, NOS2, CXCL9, and GNLY) (Figures 5A and S3A). These mRNAs were downregulated in patients with distant metastasis compared to those without distant metastases (Figure 5B and S3B). Low IDO1, CXCL10, and GBP4 levels were statistically associated with an unfavorable disease‐free survival probability (Figure 5C). There were no statistically significant differences in survival between the low and high mRNA expression groups for CIITA, NOS2, CXCL9, and GNLY (Figure S3C). We also found that the expression of the three LINC01871‐related mRNAs, namely, IDO1, CXCL10, and GBP4, was highly negatively correlated with TNM stage, lymph node metastasis, and liver metastasis (Table 3). In addition, CIITA and GNLY were identified as significant prognostic factors based on the chi square test, as shown in Table S5, and GNLY expression was highly negatively correlated with TNM stage, lymph node metastasis, and distant metastases (*p* = 0.026, 0.042 and 0.005, respectively), indicating that lower GNLY expression was related to a poorer outcome for CRC patients in the TCGA dataset. Taken together, these findings identified IDO1, CXCL10, GBP4, CIITA, and GNLY as hub LINC01871‐related mRNAs based on the DFS analysis and chi square test results. Based on these findings, a reduction in or loss of the expression the four AP002498.1‐related mRNAs and five LINC01871‐related mRNAs was determined to be associated with a decreased survival time.

### 3.5 Prediction and construction of ceRNA axes

TargetScan was used to predict upstream miRNAs of the coexpressed mRNAs and mRNA–miRNA interaction pairs to construct the ceRNA axes for AP002498.1 and LNC01871. The numbers of miRNAs potentially interacting with SERPINA1, ITLN1, REG4, L1TD1, IGFALS, and MUC5B were 889, 775, 251, 337, 70, and 306, respectively. In addition, the numbers of candidate miRNAs of CIITA, CXCL9, CXCL10, GBP4, GNLY, IDO1, and NOS2 were 2650, 906, 292, 1449, 112, 179, and 224, respectively.

miRDB (http://www.mirdb.org/) was used to predict the lncRNA–miRNA pairs associated with AP002498.1 and LNC01871. Moreover, correlation analysis was performed on the key lncRNAs AP002498.1 and LNC01871 to construct ceRNA axes based on the predictions of TargetScan and miRDB. The analysis identified the AP002498.1/miR‐4443/SERPINA1, LINC01871/miR‐4644 and miR‐185‐5p/GNLY axes as prognostic biomarkers for distant metastasis in patients with CRC, indicating the predictive ability of these two distant metastasis‐related ceRNA axes.

**FIGURE 6:**
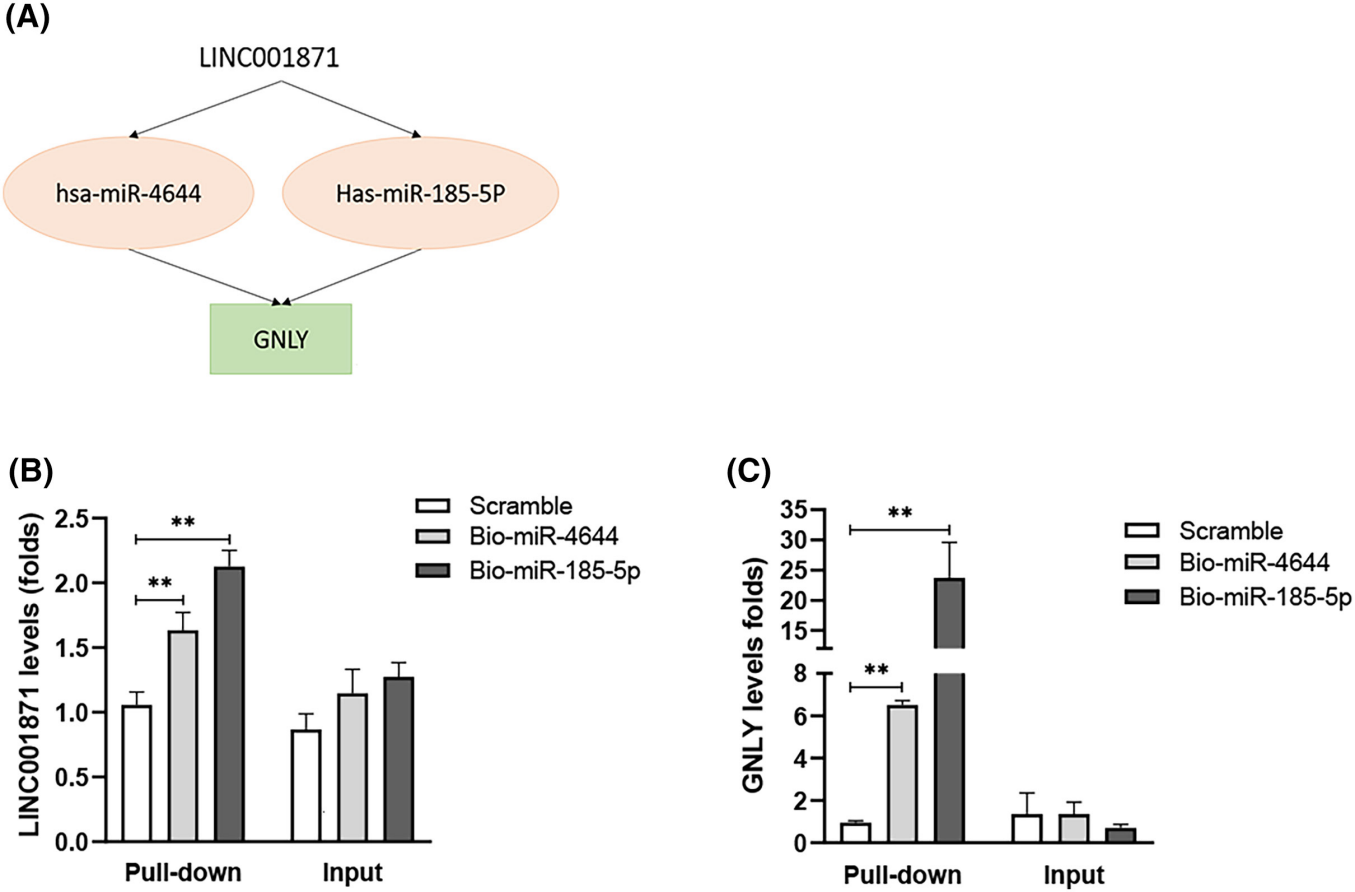
Competing endogenous RNA (ceRNA) regulatory subnetworks constructed based on LINC01871 and its coexpressed mRNAs. (A) LINC01871/miR‐4644 and miR‐185‐5p/GNLY axes. (B) Biotinylated miR‐4644 and miR‐185‐5p associate with LINC01871. (C) Biotinylated miR‐4644 and miR‐185‐5p associate with GNLY.

To detect the direct interactions in the two key lncRNA–miRNA–mRNA ceRNA axes, a biotinylated microRNA pull‐down assay was performed. The interactions between LINC01871 and miR‐4644 and between LINC01871 and miR‐185‐5p were proven, as shown in Figure 6. In addition, GNLY was indicated to be the direct target transcript of miR‐4644 and miR‐185‐5p. In contrast, only miR‐4443 was found to be regulated by AP002498.1 in the AP002498.1‐mediated ceRNA network (Figure S4).

## 4 DISCUSSION

CeRNA networks play important roles in regulating the relapse and distant metastasis of malignant tumors.^22^, ^23^, ^24^ As key upstream nodes in ceRNA networks, lncRNAs may be exploited to clarify the mechanism of CRC carcinogenesis and development and serve as molecular markers and therapeutic targets for CRC.^15^ However, there is a lack of ceRNA network research directly related to distant metastasis of CRC. In this study, we observed a very interesting phenomenon in our stratified differential expression analysis based on distant metastasis status.

Using TCGA RNA‐seq data for CRC patients, we constructed a distant metastasis‐related ceRNA network to explore the potential role of lncRNAs and their related genes in CRC diagnosis and prognosis. AP002489.1 and LINC01871 were both downregulated in CRC tissues from patients with distant metastasis. AP002498.1 is a novel OS‐related lncRNA that has not been previously reported (Figure 3 and Table S6). We identified it as an independent protective prognostic factor for CRC patients. In contrast, LINC01871 expression was significantly negatively correlated with DFS; however, it was not an independent factor. CRC patients with lower expression of LINC01871 had shorter disease‐free survival times. Based on DEmRNA coexpression analysis and miRNA predictions, we identified the ceRNA regulatory subnetworks for LINC01871. The lncRNA AP002498.1 could affect the distant metastasis and prognosis (OS) of CRC as an independent protective prognostic factor.^25^, ^26^, ^27^, ^28^ The LINC01871/miR‐4644 and miR‐185‐5p/GNLY axes might affect distant metastasis and prognosis (DFS) of CRC by regulating the radiosensitivity, ferroptosis, autophagy, and stemness of CRC cells and the tumor immune microenvironment.^27^, ^29^, ^30^, ^31^, ^32^, ^33^, ^34^, ^35^, ^36^, ^37^, ^38^

Based on the lncRNA–mRNA coexpression relationships, AP002498.1 could positively regulate SERPINA1, ITLN1, REG4, L1TD1, IGFALS, and MUC5B mRNA expression by acting as a miRNA sponge. As shown in Figure 5 and Table 2, SERPINA1, ITLN1, and REG4 upregulation in primary tumor tissues was negatively correlated with distant metastasis, indicating a protective effect on prognosis in patients with CRC. This result is consistent with the previously reported role of these three factors in other solid tumors,^25^, ^26^, ^28^, ^39^, ^40^, ^41^, ^42^, ^43^ in which they were reported to affect distant metastasis and prognosis through modulating either the stemness or autophagy of tumor cells or by regulating the immune microenvironment. In addition, miR‐4443 regulated by AP002498.1 promotes liver metastasis of breast cancer via microenvironment‐induced TIMP2 loss,^44^ and highly expressed lncRNA AP002498.1 may serve as an endogenous sponge to downregulate the expression and inhibit the function of miR‐4443.

CIITA, CXCL9, CXCL10, GBP4, GNLY, IDO1, and NOS2 were predicted to be coexpressed genes in the LINC01871 regulatory network. Studies have demonstrated that LINC01871 is involved in stemness, autophagy, ferroptosis, and the immune microenvironment in breast cancer cells; it was reported to be associated with a good prognosis and a lower incidence of distant metastasis in gastric, cervical, and endometrial cancers.^27^, ^29^, ^32^, ^37^, ^45^ In this study, LINC01871 and its coexpressed mRNAs (CIITA, CXCL10, GBP4, and IDO1) were found to be closely related to DFS rather than OS in CRC patients. Integrating these results with previous reports in the literature^27^, ^30^, ^37^, ^38^, ^39^, ^40^, ^41^, ^43^, ^44^, ^45^, ^46^, ^47^ suggested that the LINC01871‐mediated ceRNA network could affect the distant metastasis of CRC by regulating mainly the immune microenvironment rather than the stemness, autophagy, focal death, or radiosensitivity of CRC cells. Specifically, the expression of GNLY, which is regulated by LINC01871, has been associated with a reduced incidence of distant metastasis. Many studies have shown the involvement of GNLY in the targeting of tumors by cytotoxic immune cells and have revealed a correlation between the presence of granulysin and a more positive cancer prognosis.^48^, ^49^ In addition, Lei et al.^31^ found that miR‐4644 could accelerate CRC cell proliferation and migration, while Sun et al.^50^ demonstrated that miR‐185‐5p is involved in chemotherapy resistance in gastric cancer patients, suggesting the protective role of LINC01871 as a sponge of miR‐4644 and miR‐185‐5p.

Taken together, these results indicated that the lncRNA AP002498.1 and LINC01871‐associated ceRNA subnetworks affected the OS and DFS prognoses of CRC, respectively, and provided helpful insights and shed new light on areas of research for identifying diagnostic markers and therapeutic approaches for distant metastasis of CRC. Among these insights, the reversibility of the tumor immune microenvironment^47^ shows that LINC01871‐induced targeted therapy is more suitable for preventing and interfering with distant metastasis of CRC during the DFS window.

However, there are still some limitations to the present study. First, the number of patients with distant metastasis was relatively small in both the TCGA dataset and our validation cohort. Thus, a larger number of samples are needed for further analysis. Second, in vitro and in vivo verification experiments are needed to verify our hypotheses and develop novel targeted therapy for the distant metastasis of CRC.

## 5 CONCLUSIONS

In conclusion, based on the two downregulated lncRNAs in CRC tissues with distant metastasis, we identified one distant metastasis‐related lncRNA signature that could independently predict the OS of CRC patients and one that could independently predict the disease‐free survival of CRC patients. Furthermore, a potential ceRNA regulatory network was accurately constructed, suggesting implications for promising predictive factors for distant metastasis and prognosis in CRC and thus possibly providing new insight into the mechanism underlying distant metastasis of CRC.

## AUTHOR CONTRIBUTIONS

**Na Wu:** Conceptualization (equal); data curation (equal); formal analysis (equal); funding acquisition (equal); writing – original draft (equal); writing – review and editing (equal). **Jingyi Chen:** Data curation (equal); formal analysis (equal); methodology (equal). **Tingru Lin:** Methodology (equal). **Zhaohui Zhong:** Resources (equal). **Mei Li:** Methodology (equal). **Yimeng Yu:** Methodology (equal). **Jingzhu Guo:** Project administration (equal); supervision (equal); visualization (equal). **Weidong Yu:** Conceptualization (equal); data curation (equal); funding acquisition (equal); project administration (equal); writing – original draft (equal); writing – review and editing (equal).

## FUNDING INFORMATION

This research was funded by the National Natural Science Foundation of China (Nos. 32,070,116, 81,672,853).

## CONFLICT OF INTEREST STATEMENT

The authors declare no competing interests. The patent related to this work has been approved by China National Intellectual Property Administration.

## INFORMED CONSENT STATEMENT

Informed consent was obtained from all subjects involved in the study.

## Supporting information


Figure S1.

Figure S2.

Figure S3.

Figure S4.

Table S1.
Table S2.
Table S3.
Table S4.
Table S5.

Table S6.

## Data Availability

The datasets used and analyzed during the current study are available from the Cancer Genome Atlas (TCGA) (https://portal.gdc.cancer.gov/).
