# Supplementary figures and images for: Identification of AP002498.1 and LINC01871 as prognostic biomarkers and therapeutic targets for distant metastasis of colorectal adenocarcinoma
Source: Cancer Med. 2023 Dec 11;13(1):e6823. doi: 10.1002/cam4.6823 (PMC10807603; doi:10.1002/cam4.6823)

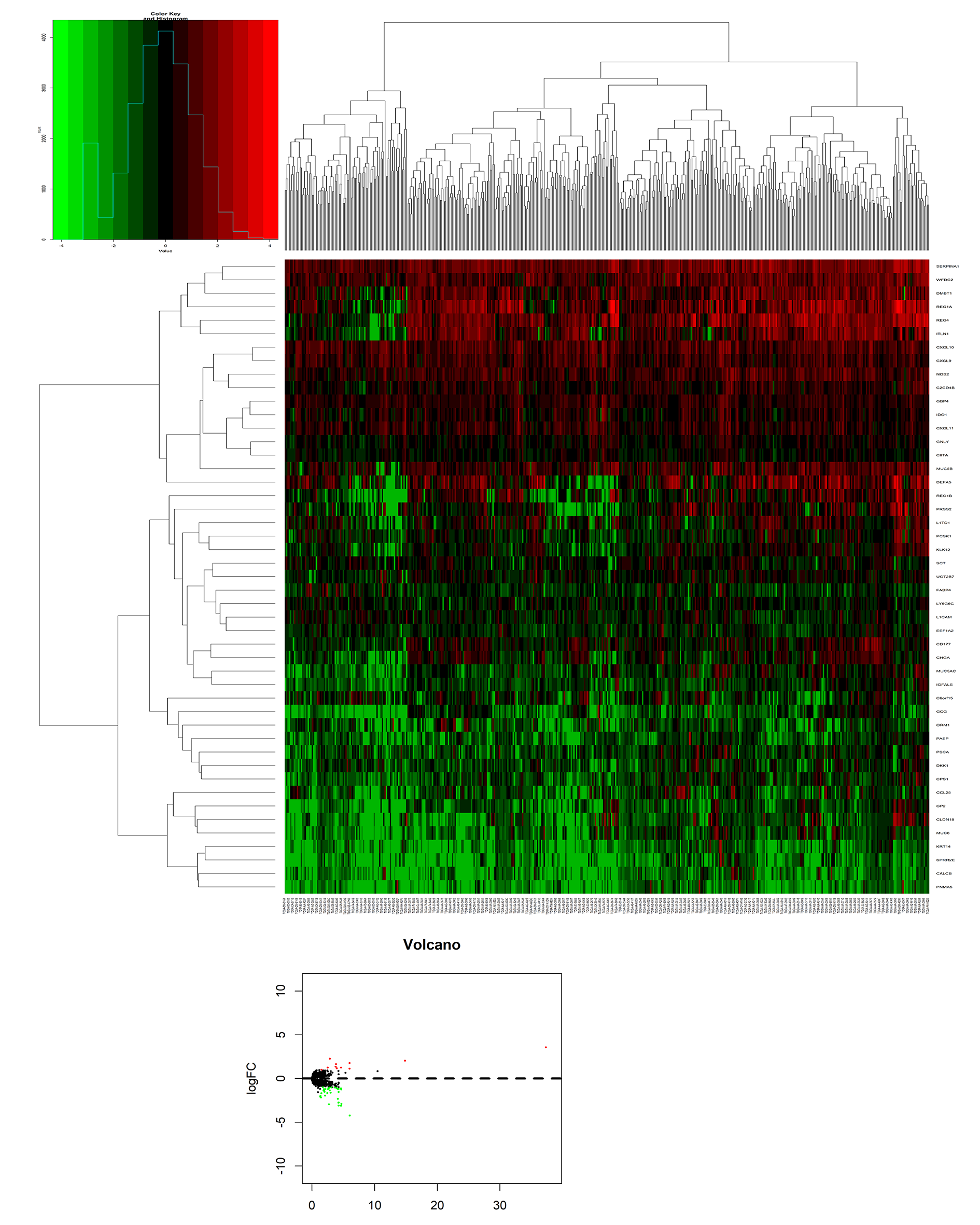

Supplement: Supplementary file 1 — Figure S1. [file CAM4-13-e6823-s006.tif]

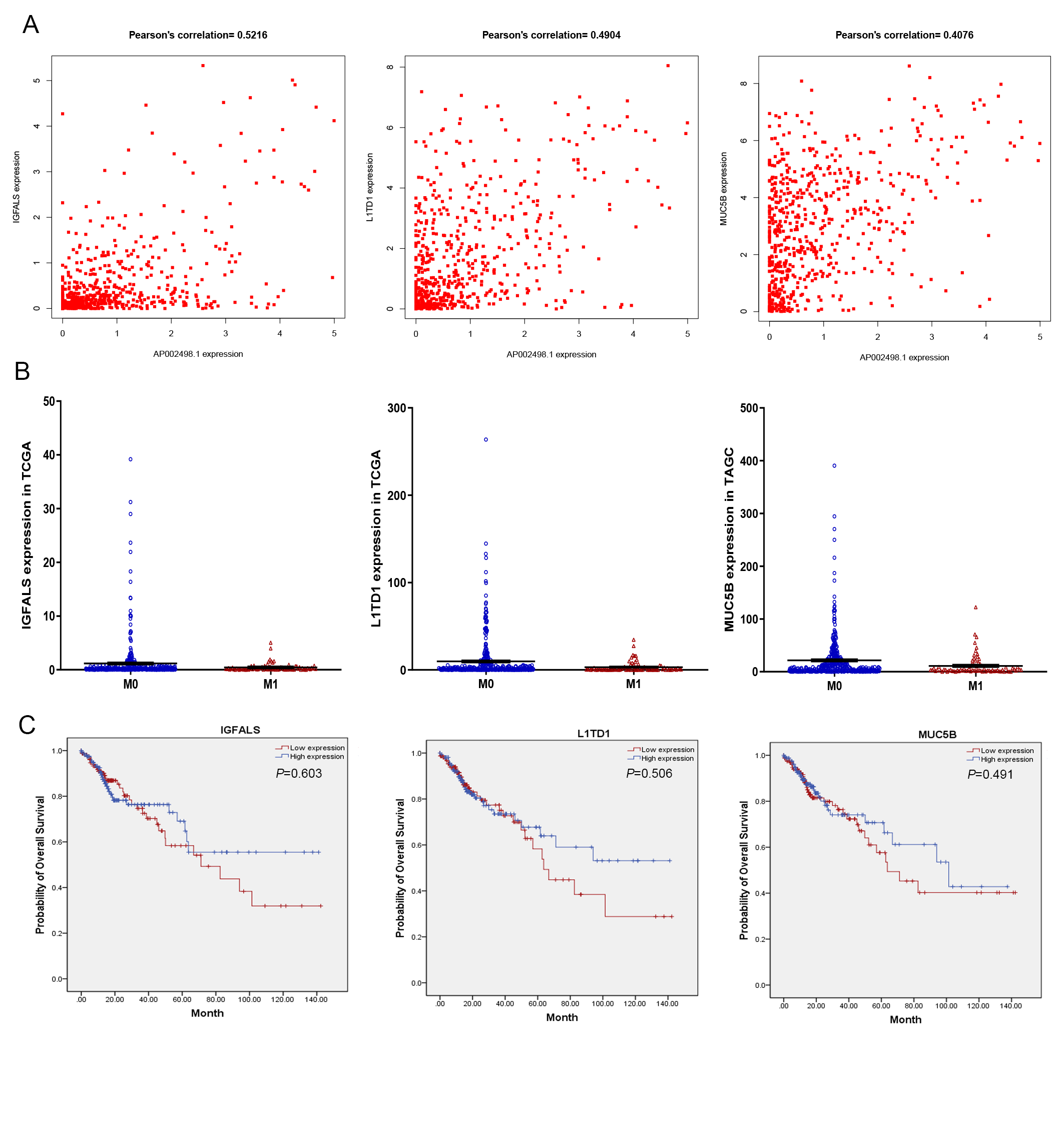

Supplement: Supplementary file 2 — Figure S2. [file CAM4-13-e6823-s001.tif]

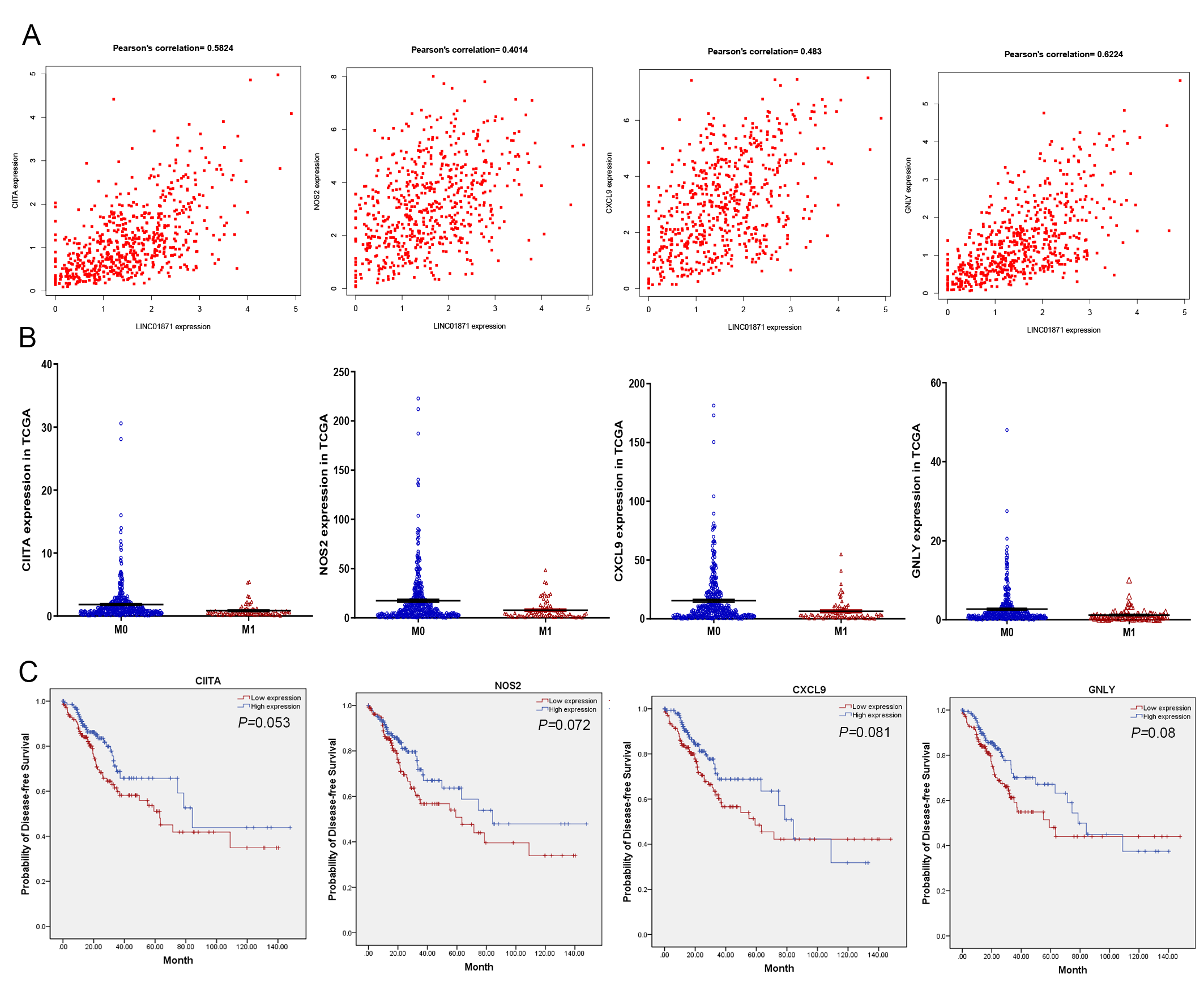

Supplement: Supplementary file 3 — Figure S3. [file CAM4-13-e6823-s003.tif]

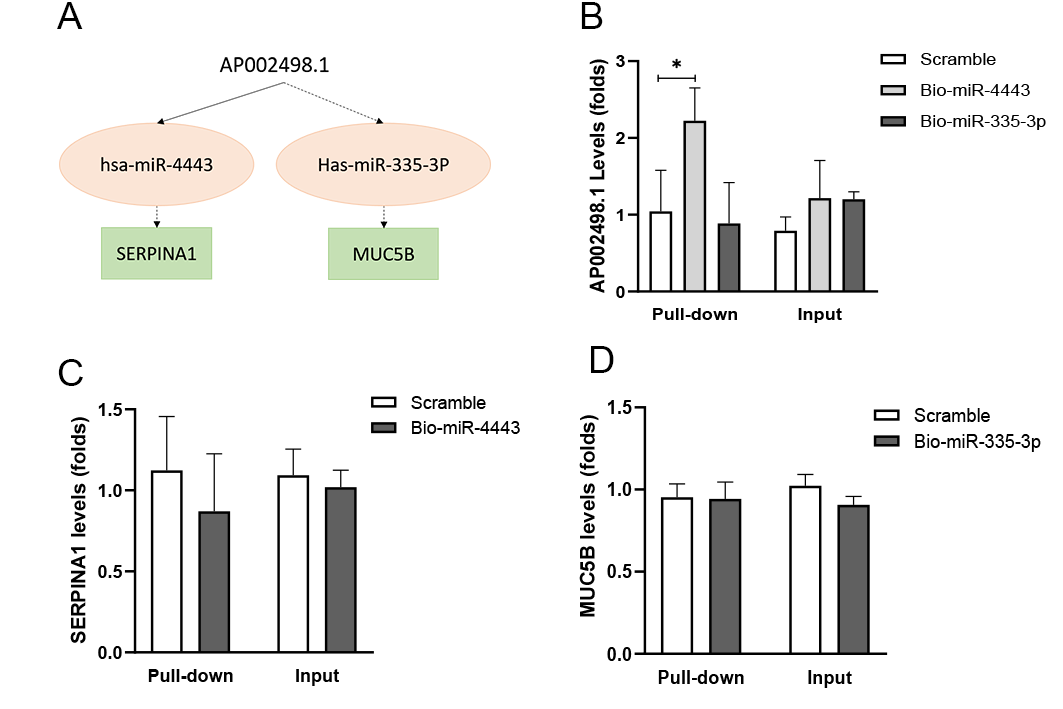

Supplement: Supplementary file 4 — Figure S4. [file CAM4-13-e6823-s005.tif]
