# Supplementary material for: Identification of AP002498.1 and LINC01871 as prognostic biomarkers and therapeutic targets for distant metastasis of colorectal adenocarcinoma
Source: Cancer Med. 2023 Dec 11;13(1):e6823. doi: 10.1002/cam4.6823 (PMC10807603; doi:10.1002/cam4.6823)
Supplement: Supplementary file 5 — Table S1. Table S2. Table S3. Table S4. Table S5. [file CAM4-13-e6823-s004.docx]

# Supplementary Tables

Table S1. The Clinicopathological parameters of 46 cases

| Clinicopathological  parameter | Numeber of cases | Percentage |
| --- | --- | --- |
| Total | 46 |  |
| Age |  |  |
| <65 | 13 | 28.3% |
| ≥65 | 33 | 71.7% |
| Sex |  |  |
| Male | 25 | 54.3% |
| Female | 21 | 45.7% |
| TNM stage |  |  |
| Ⅰ | 8 | 17.4% |
| Ⅱ | 16 | 34.8% |
| Ⅲ | 16 | 34.8% |
| Ⅳ | 6 | 13.0% |
| Tumor stage |  |  |
| T1+T2 | 9 | 19.6% |
| T3+T4 | 37 | 80.4% |
| Lymph node metastasis |  |  |
| Negative | 26 | 56.5% |
| Positive | 20 | 43.5% |
| Liver Metastases |  |  |
| Negative | 40 | 87.0% |
| Positive | 6 | 13.0% |

Table S2. The Clinicopathological parameters of 548 cases from TCGA

| Clinicopathological  parameter | Numeber of cases | Percentage |
| --- | --- | --- |
| Total | 548 |  |
| Age |  |  |
| <65 | 218 | 39.8% |
| ≥65 | 330 | 60.2% |
| Sex |  |  |
| Male | 292 | 53.3% |
| Female | 256 | 46.7% |
| TNM stage |  |  |
| Ⅰ | 96 | 17.5% |
| Ⅱ | 210 | 38.3% |
| Ⅲ | 149 | 27.2% |
| Ⅳ | 78 | 14.2% |
| Unknow | 15 | 2.7% |
| Tumor stage |  |  |
| T1+T2 | 111 | 20.3% |
| T3+T4 | 436 | 79.6% |
| Tis | 1 | 0.2% |
| Lymph node metastasis |  |  |
| Negative | 323 | 58.9% |
| Positive | 224 | 40.9% |
| NX | 1 | 0.2% |
| Liver Metastases |  |  |
| Negative | 408 | 74.5% |
| Positive | 77 | 14.1% |
| MX | 55 | 10.0% |
| Unknow | 8 | 1.5% |

Table S3. Primer information

| lncRNA | Forward primer | Reverse primer | Product length |
| --- | --- | --- | --- |
| AP002498.1 | GGAAGCACCAGGATCCCATTC | GTCTTCTCAGGTCACTGTCCA | 95 bp |
| LINC01871 | GTCACACTACCAAAGGCCG | GAGAGACAGATCGTCCACGG | 93 bp |
| BX322234.2 | GCCATTTGTTCCCACTGAGC | TCTAAGGCCTGGAATGCACC | 236 bp |
| H19 | TACTTCCTCCACGGAGTCGG | CTGCTGTTCCGATGGTGTCT | 192 bp |
| LINC00261 | AGGATTCTGCATGTGGGTGG | GGTGAGCCCCAGCCTTATTT | 275 bp |
| AC026336.3 | TCAGAGTCCATGCGAGAGGA | TCTACCACCGGGAAAATGCC | 217 bp |

Table S4. Correlations between clinicopathological parameters and three DEmRNAs co-expressed with AP002498.1 in TCGA dataset (IGFALS, L1TD1, and MUC5B).

| Clinicopatho-logical  parameter | No. | L1TD1  level | Chi-square | *P* value | IGFAL  level | Chi-square | *P* value | MUC5B level | Chi-square | *P* value |
| --- | --- | --- | --- | --- | --- | --- | --- | --- | --- | --- |
|  |  | Low/High |  |  | Low/High |  |  | Low/High |  |  |
| Total | 325 | 163/162 |  |  | 163/162 |  |  |  |  |  |
| Age | 325 |  |  |  |  |  |  |  |  |  |
| <65 | 159 | 81/78 | 0.078 | 0.78 | 79/80 | 0.027 | 0.87 | 83/76 | 0.55 | 0.47 |
| ≥65 | 166 | 82/84 |  |  | 84/82 |  |  | 80/86 |  |  |
| Sex | 325 |  |  |  |  |  |  |  |  |  |
| Male | 178 | 100/78 | 5.71 | 0.017* | 97/81 | 2.56 | 0.11 | 88/90 | 0.081 | 0.78 |
| Female | 147 | 63/84 |  |  | 67/80 |  |  | 75/72 |  |  |
| TNM stage | 311# |  |  |  |  |  |  |  |  |  |
| 0-II | 173 | 85/88 | 0.28 | 0.59 | 92/81 | 1.13 | 0.29 | 79/94 | 3.15 | 0.076 |
| III-IV | 138 | 72/66 |  |  | 65/73 |  |  | 77/61 |  |  |
| Tumor stage | 325 |  |  |  |  |  |  |  |  |  |
| 0-II | 57 | 25/32 | 1.10 | 0.30 | 32/25 | 0.99 | 0.32 | 29/28 | 0.014 | 0.90 |
| III-IV | 268 | 138/130 |  |  | 131/137 |  |  | 134/134 |  |  |
| Lymph node metastasis status | 324# |  |  |  |  |  |  |  |  |  |
| Negative | 185 | 90/95 | 0.32 | 0.58 | 95/90 | 0.32 | 0.58 | 85/100 | 2.84 | 0.092 |
| Positive | 139 | 72/67 |  |  | 67/72 |  |  | 77/62 |  |  |
| Distant  Metastasis  status | 265# |  |  |  |  |  |  |  |  |  |
| Negative | 222 | 108/114 | 1.30 | 0.26 | 118/104 | 2.67 | 0.10 | 108/114 | 3.91 | 0.048* |
| Positive | 43 | 25/18 |  |  | 17/26 |  |  | 28/15 |  |  |

#Clinical data missing, unreliable or loss to follow up, ***P*<0.01，**P*<0.05

Table S5. Correlations between clinicopathological parameters and four DEmRNAs co-expressed with LINC01871 in TCGA dataset (CIITA, NOS2, CXCL9, and GNLY).

| Clinicopatho-logical  parameter | No. | CIITA  level | Chi-square | *P* value | NOS2  level | Chi-square | *P* value | CXCL9  level | Chi-square | *P* value | GNLY  Level  Low/High | Chi-square | *P* value |
| --- | --- | --- | --- | --- | --- | --- | --- | --- | --- | --- | --- | --- | --- |
|  |  | Low/High |  |  | Low/High |  |  | Low/High |  |  |  |  |  |
| Total | 325 | 163/162 |  |  |  |  |  |  |  |  |  |  |  |
| Age | 325 |  |  |  |  |  |  |  |  |  |  |  |  |
| <65 | 159 | 83/76 | 0.52 | 0.47 | 86/73 | 1.93 | 0.17 | 86/73 | 1.93 | 0.17 | 92/67 | 7.40 | **0.007*** |
| ≥65 | 166 | 80/86 |  |  | 77/89 |  |  | 77/89 |  |  | 71/95 |  |  |
| Sex | 325 |  |  |  |  |  |  |  |  |  |  |  |  |
| Male | 178 | 90/88 | 0.026 | 0.87 | 82/96 | 2.63 | 0.11 | 88/90 | 0.081 | 0.78 | 87/91 | 0.26 | 0.61 |
| Female | 147 | 73/74 |  |  | 81/66 |  |  | 75/72 |  |  | 76/71 |  |  |
| TNM stage | 311# |  |  |  |  |  |  |  |  |  |  |  |  |
| 0-II | 173 | 83/90 | 0.98 | 0.32 | 89/84 | 0.016 | 0.9 | 84/89 | 0.26 | 0.61 | 77/96 | 4.98 | **0.026*** |
| III-IV | 138 | 74/64 |  |  | 70/68 |  |  | 71/67 |  |  | 79/59 |  |  |
| Tumor stage | 325 |  |  |  |  |  |  |  |  |  |  |  |  |
| 0-II | 57 | 28/29 | 0.029 | 0.86 | 29/28 | 0.014 | 0.90 | 35/22 | 1.61 | 0.2 | 31/26 | 0.50 | 0.48 |
| III-IV | 268 | 135/133 |  |  | 134/134 |  |  | 128/140 |  |  | 132/136 |  |  |
| Lymph node metastasis | 324# |  |  |  |  |  |  |  |  |  |  |  |  |
| Negative | 185 | 88/97 | 1.02 | 0.31 | 92/93 | 0.058 | 0.81 | 90/95 | 0.32 | 0.58 | 83/102 | 4.55 | **0.033*** |
| Positive | 139 | 74/65 |  |  | 71/68 |  |  | 72/67 |  |  | 79/60 |  |  |
| Distant  Metastasis  status | 265# |  |  |  |  |  |  |  |  |  |  |  |  |
| Negative | 222 | 104/118 | 9.19 | **0.002*** | 110/112 | 1.72 | 0.19 | 102/120 | 5.30 | 0.21 | 105/117 | 8.87 | **0.003*** |
| Positive | 43 | 31/12 |  |  | 26/17 |  |  | 28/15 |  |  | 31/12 |  |  |

#Clinical data missing, unreliable or loss to follow up, ***P*<0.01，**P*<0.05
